# Thermotropic Liquid-Crystalline and Light-Emitting Properties of Poly(pyridinium) Salts Containing Various Diamine Connectors and Hydrophilic Macrocounterions †

**DOI:** 10.3390/polym11050851

**Published:** 2019-05-10

**Authors:** Tae Soo Jo, Haesook Han, Pradip K. Bhowmik, Benoît Heinrich, Bertrand Donnio

**Affiliations:** 1 Department of Chemistry and Biochemistry, University of Nevada at Las Vegas, 4505 Maryland Parkway, Box 454003, Las Vegas, NV 89154, USA; chotaesoo@hotmail.com (T.S.J.); hanh3@unlv.nevada.edu (H.H.); 2 Institut de Physique et Chimie des Matériaux de Strasbourg (IPCMS), UMR 7504, CNRS-Université de Strasbourg, BP 43, 23 rue de Loess, F-67034 Strasbourg CEDEX 2, France; benoit.heinrich@ipcms.unistra.fr (B.H.); bdonnio@ipcms.unistra.fr (B.D.)

**Keywords:** poly(pyridinium) salts, macrocounterion, thermotropic liquid-crystalline phase, polymeric ionic liquids (PILs)

## Abstract

A set of poly(pyridinium) salts containing various diamine moieties, as molecular connectors, and poly(ethyleneglycol)-4-nonylphenyl-3-sulfopropyl ether, thereafter referred to as “Macroion”, as the hydrophilic counterion, were prepared by metathesis reaction from the respective precursory tosylated poly(pyridinium)s in methanol. The structure of these ionic polymers was established by spectroscopy and chromatography techniques. The shape-persistent ionic poly(pyridinium) materials, inserting rigid or semi-rigid diamine spacers, display thermotropic liquid-crystalline properties from room-temperature up to their isotropization (in the temperature range around 160–200 °C). The nature of the LC phases is lamellar in both cases as identified by the combination of various complementary experimental techniques including DSC, POM and variable-temperature SAXS. The other polymers, inserting bulky or flexible spacers, only form room temperature viscous liquids. These new macromolecular systems can then be referred to as polymeric ionic liquid crystals (PILCs) and or polymeric ionic liquids (PILs). All the ionic polymers show excellent thermal stability, in the 260–330 °C temperature range as determined by TGA measurements, and a good solubility in common organic solvents as well as in water. Their optical properties were characterized in both solution and solid states by UV−Vis and photoluminescent spectroscopies. They emit blue or green light in both the states and exhibit a positive solvatochromic effect.

## 1. Introduction

Polymeric ionic liquids (PILs) or polysalts are a class of polymers that combine the remarkable properties of ionic liquids (ILs) with the macromolecular properties (mechanical, durability, stability and macromolecular architecture) of polymeric materials. PILs can be prepared by polymerization of an ionic liquid monomer (anionic or cationic) leading to linear covalent backbones bearing ionic pending groups [1,2,3,4]. They usually possess physico-chemical properties that differ profoundly from those of their electrically neutral counterparts. Charge numbers, types of anions and/or cations have determining structural roles, for instance, on the polymer conformations, and on the various supramolecular assembly abilities. Their unique properties, liquid at room-temperature, high thermal and solvent stabilities make them useful for many potentially versatile applications of technological (electrical conductivity, proton exchange membrane fuel cells, solid ionic conductor, lithium-ion batteries, dye-sensitized solar cells, processing, precursor for carbon materials, porous polymers, et al.), industrial (sanitation, surfactants, detergents, powerful dispersants and stabilizers, absorbents, et al.), environmental (water treatment, soil remediation, et al.) and biological (naturally occurring biopolymers, biocompatible implants, drug delivery, et al.) relevancies [2,3,4,5,6,7,8].

In contrast, water-soluble and room temperature liquid main-chain polymers are much less common than their side-groups polymeric counterparts. The introduction of charged functional groups in the polymer main-chain may represent a successful strategy to address these issues [9]. The combination of main-chain polymer and ionic functionality results in new materials that can be used for biosensors [10,11,12,13,14,15], microelectronic or photovoltaic devices [16]. For example, p-conjugated polymers such as polythiophenes [17,18,19], polyfluorenes [20,21,22,23], and poly(phenylenevinylene)s [24] can be made soluble in water by incorporation of amino acid, ammonium or imidazolium groups; furthermore, these substituted polymers can have specific metal ion recognition which can be used in drug delivery and cell imaging.

Among the numerous examples of ionic polymers found in literature, a class of particularly promising main-chain cationic polymers are the poly(pyridinium) salts. In particular, the synthesis of poly(pyridinium) salts with two *N*-aryl pyridinium salts separated by 1,4-phenylene connectors has been recently developed and studied by Harris et al. [25,26] and Bhowmik et al. [27,28,29,30,31,32,33,34,35,36,37,38,39,40], respectively. Their preparation involves the ring-transmutation reaction of bispyrylium salts with aromatic, heterocyclic or aliphatic diamines in dimethyl sulfoxide (DMSO) at elevated temperatures. This method usually provides high-molecular-weight polymers, as supported by their inherent large viscosity measurements and gel-permeation chromatography. In addition, this method has been successfully applied to structurally related systems incorporating larger aromatic spacers as reported by Mikroyannidis et al. [41]. Depending on both the structure of the backbones and the nature of the counterion, such polymers demonstrate good solubility in protic solvents, but not in water, may additionally display lyotropic phases in various polar organic solvents or thermotropic properties, and also emit UV/visible light both in solutions of polar organic solvents and in the solid state.

**Scheme 1:**
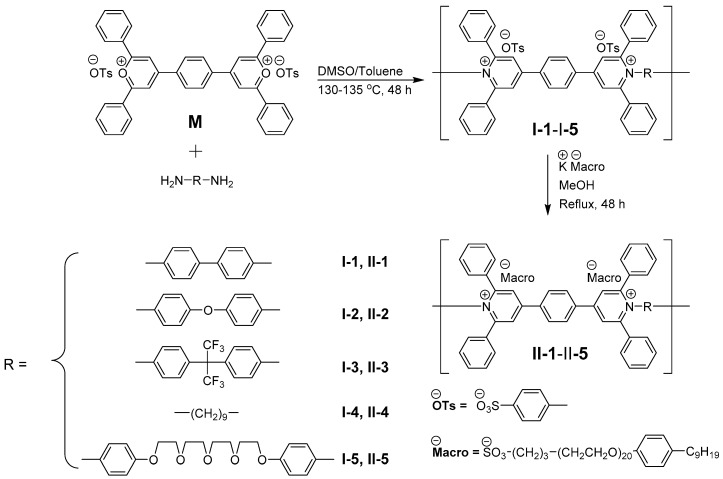
Synthesis and molecular structures of water-soluble poly(pyridinium) salts with macrocounterion, **II-1**–**II-5.**

Herein, we report on the synthesis of a series of room-temperature liquid, liquid-crystalline and water-soluble poly(pyridinium) salts having bulky macrocounterions (Scheme 1) and rigid, semi-rigid, or flexible aromatic/aliphatic diamine moieties in the polymeric main-chains. Irrespective of the backbones, all the polymeric chains are water-soluble. Furthermore, the introduction of the macrocounterion within the polymeric structure not only provides water-soluble poly(xylidene) precursor to poly(phenylene vinylene) but also thermal elimination of tetrahydrothiophene in air at low temperature (ca. 80 °C) to give defect-free luminescent films [42,43]. The ion exchange from tosylate to macroanion also affords the induction of nearly room temperature thermotropic mesophases for the most rigid polymeric systems. Their chemical structures, thermal behavior, including thermotropic liquid-crystalline (LC) behavior, and optical properties have been characterized by using several complementary experimental techniques. Light-emitting properties in several organic solvents and water as well as in the solid state have been also examined by photoluminescence spectrometry. The chemical structures and designations of ionic polymers, which were prepared and characterized in this study, are shown in Scheme 1.

## 2. Materials and Methods

Benzidine, 2,2′-bis(4-aminophenyl)hexafluoropropane, nonane-1,9-diamine and poly(ethylene glycol) 4-nonylphenyl 3-sulfopropyl ether potassium salt (macrosalt) were purchased from Sigma Aldrich (Milwaukee, WI, USA) and used as received; 4,4′-oxydianiline was purchased from TCI America and purified by sublimation before use. The monomer 4,4′-(1,4-phenylene)bis(2,6-diphenylpyrylium)ditosylate, **M**, was synthesized according to the reported procedure [27]. Bis(2-(2-(4-aminophenoxy)ethoxy)ethyl) ether was synthesized according to the reported procedures [35,44]. Common organic solvents were purchased from commercial vendors (Sigma Aldrich (Milwaukee, MI, USA), Alfa Aesar (Ward Hill, MA, USA), Acros Organics (Morris, NJ, USA), and TCI America (Portland, OR, USA) and used without further purification.

### 2.1. General Procedure for the Synthesis of Polymers I-1–I-5

The bis(pyrylium) tosylate salt, **M**, was reacted with an appropriate diamine monomer, H_2_N-**R**-NH_2_ (Scheme 1), by a ring-transmutation reaction to yield each of the targeted polymers. Equal mole ratios of the two monomers dissolved in DMSO were introduced in a three-necked round bottom flask equipped with a magnetic stirrer. A small amount of toluene was added to remove the water generated during the reaction by azeotropic distillation, which was facilitated by using a Dean-Stark trap and water condenser. The temperature was increased up to 130–135 °C and the solution was stirred for 48 h under inert nitrogen atmosphere. After the polymerization was completed, the solution was cooled down to room temperature. Excess of DMSO was removed by a rotary evaporator until the solution became viscous, which was then poured into water to allow the polymer to precipitate. The solid residue was washed with copious amounts of water to remove any residual impurities and collected by vacuum filtration. The crude products were additionally washed a few times with boiling water and dried in vacuo at 110 °C for 48 h and yield the polymers (**I-1**–**I-5**) in good yields.

### 2.2. General Procedure for the Synthesis of Polymers II-1–II-5

Polymers **II-1**–**II-5** were prepared by metathesis reaction from the respective tosylated polymers (**I-1**–**I-5**) with an excess of macroion potassium salt in common solvents. Typically, polymer **I-1** (500 mg) was dissolved in methanol (25 mL) and DMSO (a few drops) and macrocounterion (2.1 equiv based on polymer repeating unit molar ratio) was added into the round-bottomed flask. The reaction mixture was heated to reflux for 48 h with stirring. Then, the excess of methanol was removed by a rotary evaporator to yield a viscous liquid, and diethyl ether was poured to precipitate the polymer. The metathesis step was repeated once or twice until all the tosylate counterion was completely exchanged by the macroion, as monitored by ^1^H NMR spectroscopy. The collected gel-like polymer was washed with diethyl ether to remove any remaining excess of macroions. The washed polymer was dried in vacuo at 80 °C for 24 h.

### 2.3. Analytic, Spectroscopic, Thermal and Structural Polymers Characterization

FTIR spectra were recorded with a Nicolet FTIR analyzer with their neat films on KBr pellets. ^1^H and ^13^C NMR spectra of the ionic polymers were obtained using a Varian NMR spectrometer (400 MHz for ^1^H and 100 MHz for ^13^C) with three RF channels at room temperature, and chemical shifts were referenced to tetramethylsilane (TMS). NMR samples were prepared on gentle heating to dissolve the polymer in *d_6_*-DMSO. The ^1^H and ^13^C NMR samples were prepared at a concentration of 10 and 30 mg/mL of polymer, respectively. For polymers **I-1, I-2** and **I-5**, gel permeation chromatography (GPC) analysis was conducted using a VISCOTEK chromatograph equipped with three ViscoGel1-MBHMW-3078 columns and a tetra detector array (UV/visible, low and right angle light scattering, refractive index, and viscometer) at 50 °C. DMSO containing 0.1 M LiBr as the mobile phase and the flow rate was set at 1.0 mL/min. The instrument was calibrated using pullulan standard of P-50. Weight average molecular weights, *M*_w_, were determined using the light scattering detector using the OmniSEC software. The instrument was calibrated using a Pullulan P-50 standard. Differential scanning calorimetry (DSC) measurements were conducted on TA module DSC Q200 series in nitrogen at heating and cooling rates of 10 °C/min. The temperature axis of the DSC thermograms was calibrated before using the reference standard of high purity indium and tin. Thermogravimetric analysis (TGA) was performed using a TGA Q50 instrument in nitrogen; the TGA data were collected at temperatures between 30 and 500 °C at a heating rate of 10 °C/min. The thermotropic LC properties of the ionic polymers were determined using polarized optical microscopy (POM, Nikon, Model Labophot 2) equipped with a crossed polarizer. Small amounts of the polymers were placed between two cover glasses and then heated above the melting transition up to the isotropization temperature and cooled down slowly (typically 1–2 °C/min). The temperature-variable small-angle X-ray scattering (SAXS) measurements of polymers **II-1**–**II-5** were carried out on samples on both heating and cooling cycles, with temperatures steps of 20 and 10 °C, respectively. Samples were exposed to an X-ray beam for about one to two hours at these temperatures. The SAXS patterns were obtained using the following setup. A linear monochromatic CuK_α1_ beam (λ = 1.5405 Å) was obtained using a sealed-tube generator (900 W) equipped with a bent quartz monochromator. The crude powder was filled in Lindemann capillary of 1 mm diameter and 10 μm wall thickness. The diffraction patterns were recorded with a curved INEL CPS120 counter gas-filled detector linked to a data acquisition; periodicities up to 80 Å can be measured, and the sample temperature can be controlled to within ±0.05 °C from 20 to 200 °C. The UV−Vis absorption spectra of polymer solutions in organic solvents were recorded at room temperature using Varian Cary 50 Bio UV−Visible spectrophotometer in quartz cuvettes. Photoluminescence spectra in various solutions and thin films were recorded with a PerkinElmer LS 55 luminescence spectrometer with a xenon lamp light source.

## 3. Results and Discussion

### 3.1. Chemical Structures

**Figure 1:**
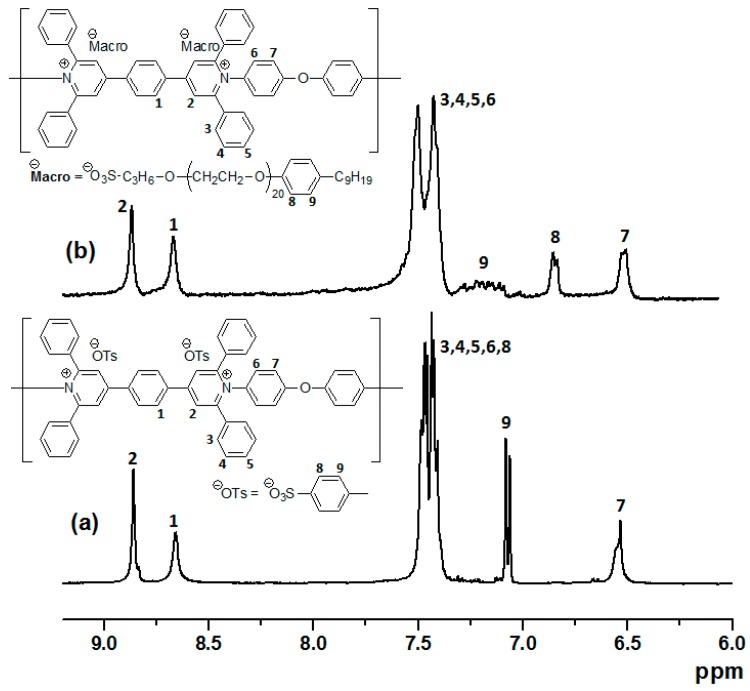
Expanded aromatic regions of ^1^H NMR spectra of (a) **I-2** and (b) **II-2** (10 mg/mL in *d_6_*-DMSO).

The synthetic route for the preparation of water-soluble poly(pyridinium salt)s **II-1**–**II-5**, from the tosylated precursors **I-1**–**I-5**, is depicted in Scheme 1. As reported elsewhere [27,32,35], precursory polymers **I-1**–**I-5** were synthesized through a ring-transmutation polymerization reaction with the bis(pyrylium) salt, **M**, and various diamines, H_2_N-**R**-NH_2_, in DMSO. Water generated during the reaction was removed by azeotropic distillation to enhance the polymerization reaction. Each of the IR spectra showed the following major characteristic peaks, among others, establishing its chemical structure: IR (neat): ν (cm^−1^) 3058, 2925, 2853, 1619, 1596, 1558, 1495, 1448, 1392, 1199, 1120, 1053, 1012, 777, 701, 679, 568 (Figure S1). As shown in Figure 1, the ^1^H NMR spectrum of polymer **I-2** showed a unique set of resonances at δ = 8.87 and 8.67 ppm for the protons of the pyridinium moieties and a set of aromatic resonances at δ = 7.45 (overlapped) and at 7.10 ppm, and aliphatic resonance at δ = 2.05 ppm for the methyl protons of the tosylate counterions. Their hydrogen ratio matches the expected calculated values, meaning that the desired poly(pyridinium) salt was successfully synthesized. To make water-soluble poly(pyridinium) salts, the tosylate counterion were exchanged by the macrocounterion via metathesis reaction as outlined in Scheme 1. Initially, the reaction was carried out at an elevated temperature in DMSO because of the polymer had high solubility in this solvent. However, the complete metathesis reaction was not achieved in this solvent, and to accomplish a complete exchange reaction solvent was changed from DMSO to methanol. Under these conditions, after the reaction was heated to reflux for 48 h, the solvent was completely removed by rotary evaporation and excess diethyl ether was added into the gel-like polymer to remove any remained excess macrocounterion and precipitate out the desired polymer. The chemical structures of polymers **II-1**–**II-5** were confirmed from the analyses of ^1^H NMR spectra. When compared to the ^1^H NMR of polymer **I-2**, chosen as a representative example, the ^1^H NMR spectrum of polymer **II-2** revealed the complete disappearance of the doublet peaks of the aromatic tosylate counterions at δ = 7.45 (overlapped) and 7.10 ppm simultaneously with the appearance of new peaks at δ = 7.30 and 6.84 ppm associated to the aromatic protons of the macrocounterion. These results suggest that the tosylate counterions were totally exchanged by the macrocounterions. The other polymers, which had different linkage in their repeating unit, showed identical trends in their ^1^H NMR spectra (Figures S2–S5). 

### 3.2. Solubility

Good solubility of polymers in organic solvents and H_2_O can be of significant interest for their diverse applications because of their easy processing in thin film states. The solubility of polymers **II-1**–**II-5** was examined in various organic solvents and H_2_O. Essentially all the polymers are soluble in H_2_O, DMSO, CH_3_CN, CH_3_OH, acetone and THF up to a concentration of 10 mg/mL, except polymer **II-1** whose solubility in acetone and THF do not exceed a concentration of 1 mg/mL.

The enhanced solubility of these polymers was observed when compared to those of polymers **I-1**–**I-5**. The enhanced solubility is evidently caused by the introduction of flexible, bulky macrocounterions. These macroions resulted in the increased separation between polymer chains and also provided a favorable mechanism for the interactions of oligooxyethylene groups and various non-polar and polar solvents including water.

### 3.3. Molecular Weight Measurements

Molecular weight properties (M_n_, M_w_ and PDI) of the polymers **I-1, I-2** and **I-5** were determined using GPC with the interdetector signals such as refractometer, viscometer and low- and right-angle light scattering. To minimize the electrostatic interactions between polymer chains, a small amount of LiBr was used to suppress these interactions between the polymer chains and those of the polymer chains with GPC column packing material [45,46]. Their number-average molecular weight (*M*_n_) values were 27,571, 41,000 and 41,263 g/mol and PDI values were 1.49, 1.40 and 1.14, respectively. Given the nature of polymerization, their PDI values are rather narrow that are consistent with the results of other poly(pyridinium salt)s [27,28,29,30,31,32,33,34,35,36,37,38,39,40]. It is quite reasonable to assume that all of polymers **II-1**–**II-5** have respectable molecular weights to assess their solution, thermal, optical and properties without concern with regard to the effect of molecular weights on these properties.

### 3.4. Thermal Properties

**Figure 2:**
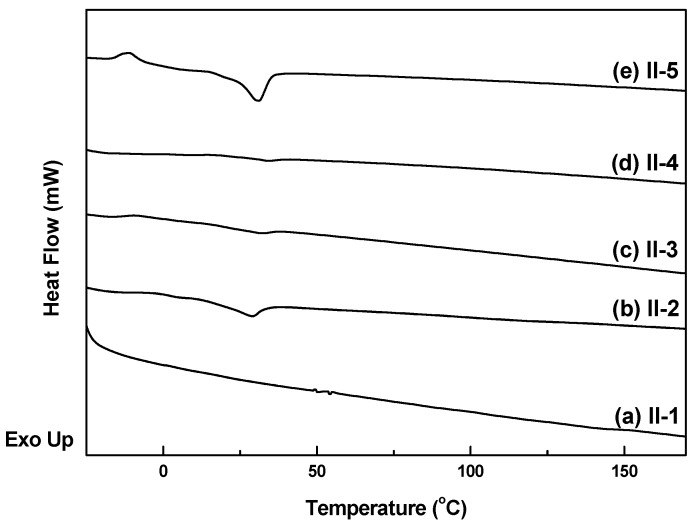
DSC thermograms of polymers **II-1**–**II-5** (second heating cycle obtained at a heating rate of 10 °C/min).

The thermal properties of polymers **II-1**–**II-5** were evaluated by DSC and TGA measurements. DSC thermograms were obtained with heating and cooling rates of 10 °C in nitrogen and are shown in Figure 2 and thermal data are compiled in Table 1. Polymer **II-5** is only marginally crystalline in the pristine state and showed cold crystallization followed by low-temperature melting to the isotropic liquid. Polymers **II-1**–**II-4** were obtained as amorphous glassy materials; **II-3** and **II-4** directly liquefy into the isotropic phase beyond their glass transition, whereas **II-1** and **II-2** show a fluid LC phase extending to high temperature. In consistency with previous results, the flexible oxyethylene linkages promote the crystalline order as they allow rigid moieties to pack in a regular way [25]. When compared to the precursory polymers bearing the tosylate ion (**I-1**–**I-4**), the polymers (**II-1**–**II-4**) show substantially lowered glass transition temperatures, *T*_g_s that may be attributed to the fact that the macroion is more flexible and bulkier than the tosylate ion. The oligo(ethylene oxide) and aliphatic segments connected to the ionic moiety indeed logically help the polymer chains to move more easily with respect to one another, which explains the lower *T*_g_s; the previous polymers **I-1** remained a solid without thermal transition up to decomposition around 350 °C, **I-2** showed a *T*_g_ at 276 °C, **I-3** at 124 °C, **I-4** at 77 °C and **I-5** at 140 °C [32,35]. The new polymers **II-1** and **II-2** liquefy to liquid crystalline phases at 40 and 29 °C, respectively, giving rise to weakly birefringent and fluid POM textures (Figure S7). These two polymers thus possess broad LC phase temperature ranges of 160 and 131 °C (Table 1), while polymers **II-3**, **II-4** and **II-5** do not form LC phase, but directly liquefy to isotropic phase at low temperatures (31–34 °C), as determined from DSC and SAXS (Figure S6). Phase transition temperatures are fatally lower with these backbones than with **II-1** and **II-2** ones since the voluminous CF_3_ groups perturb the lateral packing and chains segments add supplementary incompatibility, but further, the macroion steadily introduces further lowering and yields low-melting polymers, whose behavior is controlled by the intrinsic chemical structure of the backbones. This relatively new class of polymers can thus be considered as a new family of polymeric ionic liquids (PILs), of great relevance and huge potential in a multitude of applications [1,2,3,4,5,6,7,8]. 

**Figure 3:**
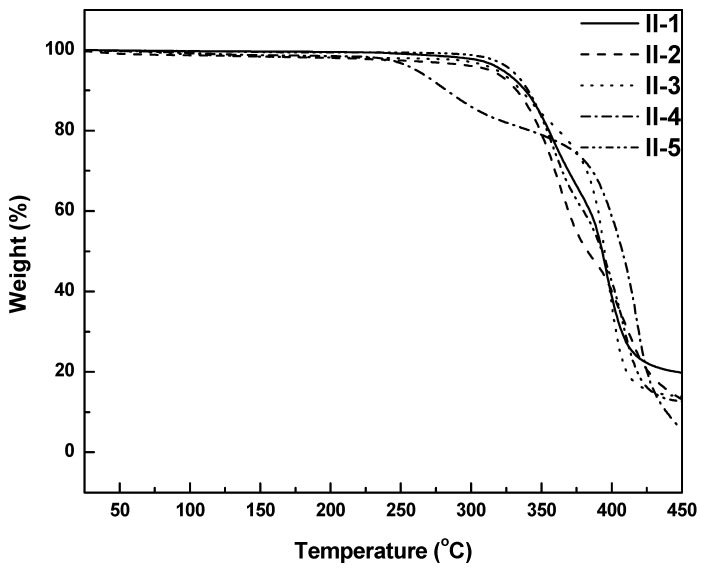
TGA thermograms of polymers **II-1**–**II-5** obtained at a heating rate of 10 °C/min in nitrogen.

Previous TGA analyses revealed that polymers **I-1**–**I-5** exhibited a two-step decomposition process (not shown). The first decomposition was found to start at a temperature around 300 ^°^C due to the cleavage of the C–S bond in the tosylate counterion, which is consistent with previously reported results for other poly(pyridinium) salts [36,37,38,39,40]. The onset of the second degradation, due to the aromatic moieties of poly(pyridinium) salts main-chain decomposition, occurred near 500 °C [26]. However, after the metathesis reaction, the decomposition temperatures (*T*_d_s) of the polymers **II-1**–**II-5**, are in most cases, increased slightly (Figure 3 and Table 1), with respect to their precursory homologues. This slight enhancement is presumably related to the higher thermal stability of the macrocounterion (i.e., degradation temperatures of sodium tosylate and potassium macro-counterion are 155 °C and ca. 350 °C, respectively). Polymer **II-4** showed the lowest *T*_d_ (261 °C) of those of the polymers in the series, which is related to the lower thermal stability of the aliphatic linkage than aromatic linkage.

### 3.5. Variable-Temperature SAXS Measurement

**Figure 4:**
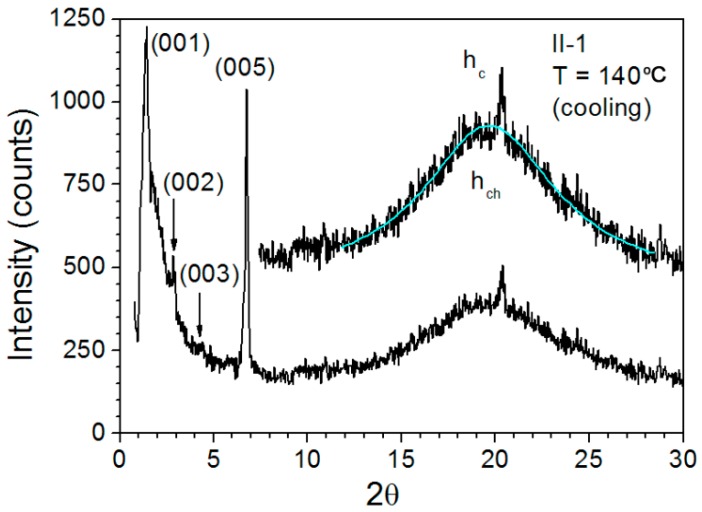
Small-angle X-ray diffraction patterns for polymer **II-1** registered on cooling at 140 °C.

**Figure 5:**
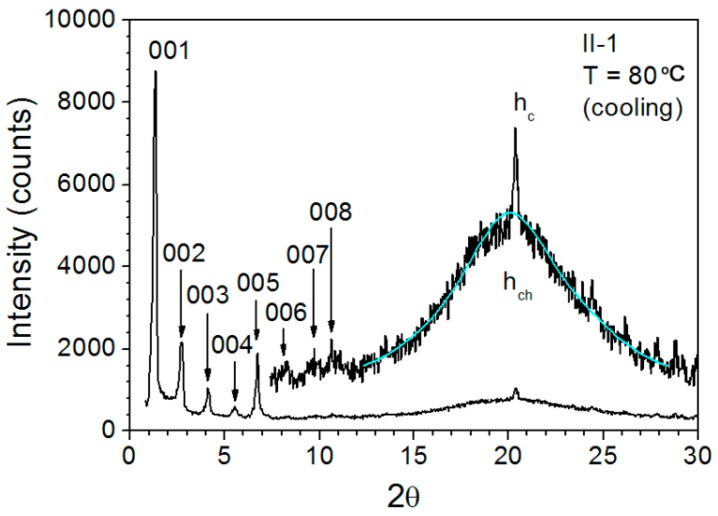
Small-angle X-ray diffraction pattern for polymer **II-1** registered on cooling at 80 °C.

**Figure 6:**
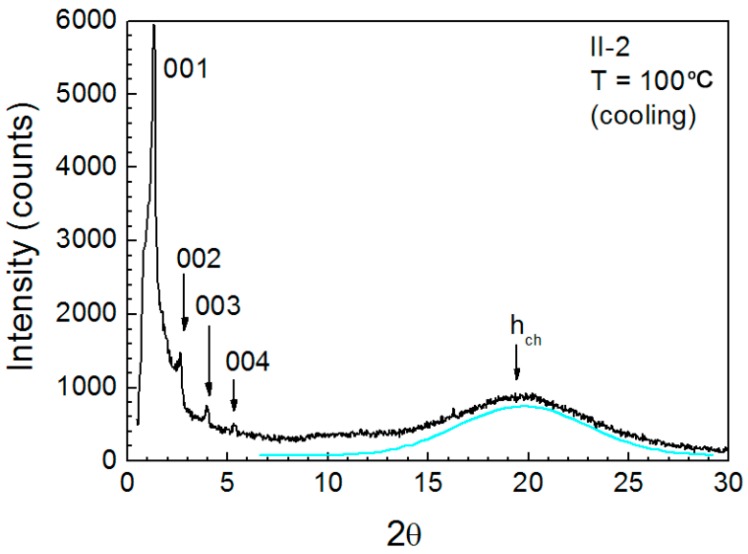
Small-angle X-ray diffraction patterns for polymer **II-2** registered on cooling at 100 °C.

To determine the nature and packing structure of the supramolecular organizations formed by the various polymers **II-1**–**II-5**, SAXS analyses were performed at different temperatures for all the polymers. SAXS experiments performed on polymers containing the bulky non-linear (**II-3**), and flexible linkages (**II-4, II-5)** confirmed their amorphous characteristics at room temperature. Only one broad scattering feature was observed in the investigated angular range, at ca 4.5 Å, that corresponds to the signal of chains and molecular parts in their molten states (Figure S6), as expected from the previous DSC and POM studies. Various subsequent thermal treatments (annealing, fast cooling, etc.) did not induce the formation of crystalline or LC states. However, as just mentioned above, the polymers **II-1** and **II-2** with rigid or semi-rigid diamine connectors, exhibit low-temperature LC phases, with a lamellar structure, as deduced from SAXS. Even though polymer **II-2** was in an amorphous state up to ca. 160 °C during the first heating cycle, an ordered lamellar structure occurred on cooling from the isotropic liquid phase, down to its transformation into an amorphous phase below 30 °C. This thermal behavior is different on the subsequent heating cycle, the long-range ordered lamellar mesophase reappearing at ca 29 °C on the second heating, the local-range molecular arrangement frozen in the glassy state is close to the mesomorphic one. Typical X-ray patterns in the lamellar phase, like the ones recorded on cooling at 140 and 80 °C for **II-1** (Figure 4 and Figure 5), and at 100 °C for **II-2** (Figure 6), exhibited a set of eight (**II-1**) to four (**II-1**, **II-2**) sharp and small-angle reflections in the ratio 1:2:3:4:5:6:7:8, 1:2:3:5 (**II-1**), and 1:2:3:4 (**II-2**), respectively, besides the diffuse scattering at 4.5 Å due to lateral distances between molten chains and rigid segments. The large number of sharp lamellar orders proves that highly segregated alternated structures were obtained and that the oligooxyethlene moiety in the macroion chain was appropriately designed. It should be mentioned that polymer **II-1** exhibits an additional high-temperature lamellar mesophase (ca. between 130–150 °C) with modified reflection intensities but nearly identical locations. In addition, both phases exhibit an intensity modulation of a similar period in the lamellar reflection series, the change in the upper phase consisting merely in the weakening of all lamellar orders except (005). These close intensity profiles reveal only minor structural re-organizations in the succession of several high electronic density strata associated with polymers and to ionic moieties. The thicknesses of these strata and the alternation with the low electronic density molten chains sublayers are thus responsible for the enhancement of the (005) order in the series, whilst the less efficient segregation and the more diffuse interfaces in the upper phase reduce the number of visible lamellar orders. The formation of separated strata rather than of mixed ionic polymer sublayers is intimately related to the sharp reflection h_c_, observed in both phases. Such a long-range in-plane periodicity is consistent with polymer chains well registered in flat sublayers separated from the surrounding flat ionic sublayers.

**Figure 7:**
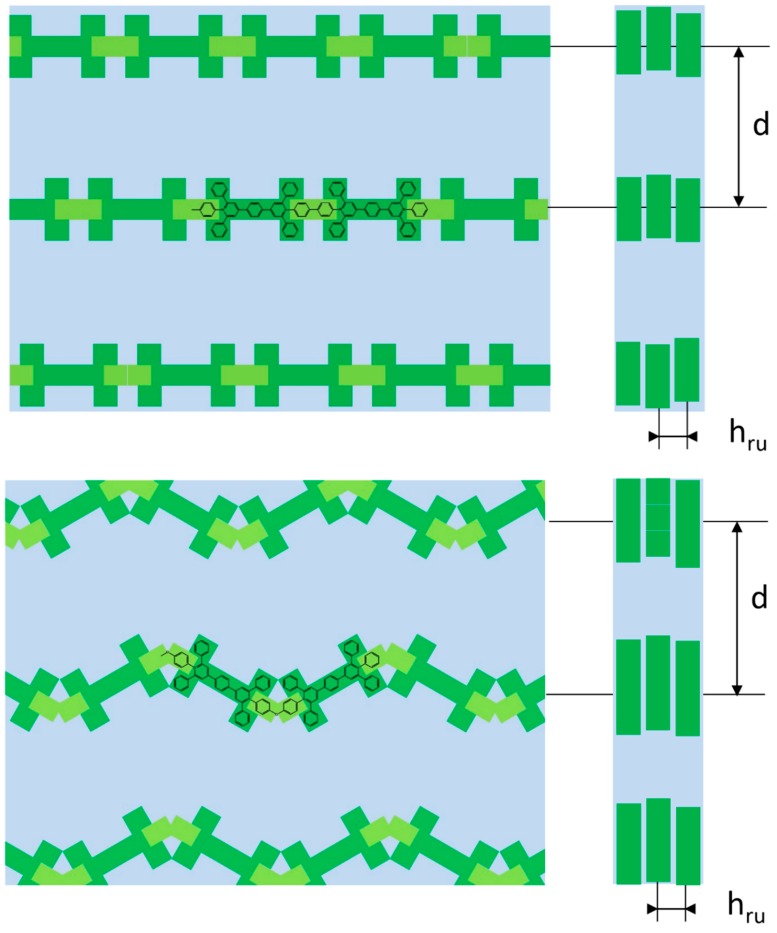
Schematic representation of the lamellar structures of both polymers **II-1** (top) and **II-2** (bottom). The blue background represents the molten pegylated and aliphatic chains of the macroion, d, the lamellar periodicity and h_ru_, the average distance between parallel polymeric backbones.

A molecular feature contributing to this regular lateral packing is the rod-like shape of the biphenyl fragment in-line and of equivalent bulkiness as the rest of the repeat unit. In contrast, the 4,4′-oxybiphenyl fragment in polymer **II-2** includes a bend, less suitable for optimized stratification in this case but not totally incompatible either, that would then explain the vanishing of the reflection h_c_ and the different intensity profile, besides the slightly increased layer thickness (Figure 4, Figure 5 and Figure 6 and Table 2). A possible representation of the lamellar ordering for both polymers is depicted in Figure 7. The absence of crossed reflections moreover shows that polymers are more or less correlated within the layers (laterally), but not between layers, where they can behave independently. Even though poly(pyridinium salt)s with various organic counterions and aliphatic or flexible aromatic diamine moieties can have thermotropic LC properties, it was not the case for those polymers with rigid or semirigid aromatic diamine moieties, with tetrafluoroborate or triflate counterions (CF_3_SO_3_−, −BF_4_) [25,26]. Polymers **I-4** and **I-5** with rigid diamine moieties and tosylate counterion developed LC texture at 220 and 155 °C, respectively [32,35], while changing the counterion to triflimide (−N(SO_2_CF_3_)_2_) reduced *T*_g_, *T*_m_ and *T*_i_ of poly(pyridinium) salts [32,35], presumably because of electrostatic interactions of lower strength in relation with the increased ion mobility and the reduced lattice energy in the crystalline state. In a similar vein, macrocounterion provided a lamellar LC phase for polymer **II-1** and **II-2** at relatively lower temperatures. These results showed that the incorporation of the bulky macrocounterion was, therefore, conducive to the thermotropic LC property of poly(pyridinium) salts due to the intercalation of interlayers of molten segments between successive ionic layers. In the case of polymers **II-3**–**II-5** with kinked, aliphatic or flexible linkages in the aromatic diamine moieties, the intercalation of the molten segments of the macroion did not preserve the regular layer alternation and LC properties but directly led to an isotropic liquid phase at low temperatures.

### 3.6. Optical Properties

**Figure 8:**
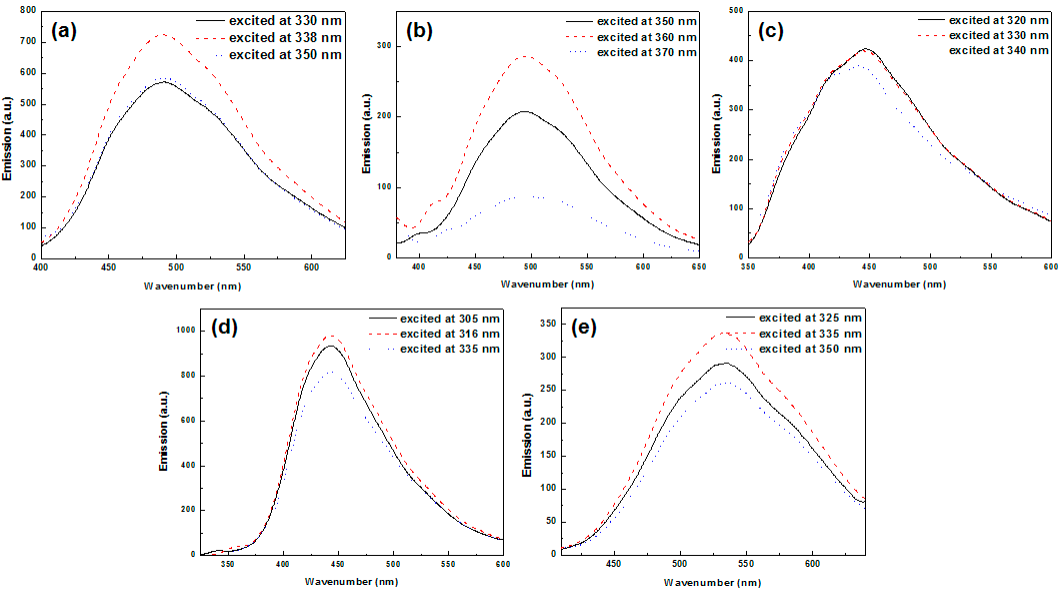
Emission spectra of polymers (**a**) **II-1**, (**b**) **II-2**, (**c**) **II-3**, (**d**) **II-4** and (**e**) **II-5** in H_2_O at various excitation wavelengths.

Due to the chromophore nature of the 4,4′-(1,4-phenylene)bis(2,6-diphenylpyridiniun) dication, incorporated in the polymeric main-chain, optical properties were studied both in solution and solid state of polymers **II-1**–**II-5** and the results are summarized in Table 3. Whatever their intimate molecular details, the polymers showed almost identical absorption maxima between 330 and 345 nm in H_2_O as detected by UV−Vis. By changing the solvents (methanol, acetone, acetonitrile, chloroform and tetrahydrofuran), no significant differences were observed suggesting closely spaced π−π* transitions. These results implied that the polarity of solvents and the interaction between solvents and polymer backbones were less sensitive to energies of the ground states of chromophores for these ionic polymers. The optical band gaps of these polymers (*E*_g_s) as determined from the onset of wavelength in the UV−Vis spectra in H_2_O were between 3.00 and 3.21 eV. These band gaps were higher than those of other conjugated polymers such as poly(*p*-phenylene vinylene)s [47,48] but comparable with other poly(pyridinium) salts [32,34,35,40]. Furthermore, the *λ*_max_ values of polymers **I-1**–**I-5** had similar absorption bands when compared to those of polymers **II-1**–**II-5** in methanol, acetonitrile, acetone, chloroform and tetrahydrofuran. The results suggested that UV−Vis spectra of these polymers were independent of the counterions (tosylate and macroion) and polarity of solvents. The PL spectra of polymers **II-1**–**II-5** in water are shown in Figure 8 and those in organic solvents are provided in Supplementary Materials (Figures S8–S12). In the case of polymer **II-1**, there was a major *λ*_em_ peak at 492 nm, along with a weak shoulder at the shorter and longer wavelength side, at an excitation wavelength of 330, 338 and 350 nm in water. In methanol, it showed a major *λ*_em_ peak at 497 nm with a shoulder peak at 523 nm when excited at wavelengths of 325, 335 and 345 nm. It showed the highest *λ*_em_ peak at 500 nm in acetonitrile. Polymer **II-2** exhibited a *λ*_em_ peak at 496 nm in water and the highest *λ*_em_ peak at 527 nm in acetonitrile. Polymers **II-3** and **II-4** emitted blue light (413-451 nm) in all examined solvents including water. In contrast, polymer **II-5** emitted green light (500-542 nm) in all examined solvents including water. The *λ*_em_ peaks for all these ionic polymers in various solvents of varying polarities are compiled in Table 3. The diverse structural parameters including rigid, semi-rigid, flexible moieties in these polymers, interactions of solvents (nonpolar, polar, hydrogen-bonded solvent, aprotic) with the polymer chains (both ground and excited) make a consistent trend in their emission spectra quite difficult to establish. However, the data in Table 3 suggest that a positive solvatochromism phenomenon is observed, i.e., the *λ*_em_ peaks were shifted to the longer wavelengths with the increase in solvent polarity [49]. The full-width at half-maximum (fwhm) values of emission spectra for polymers **II-1**–**II-5** were slightly narrow in relatively non-polar solvents such as CHCl_3_ and THF that are suggestive of isolated emitting chromophores [50].

To examine the effect of counterions, the emission spectra of polymers **I-2**–**I-5** with tosylate counterions were studied in identical solvents. As reported earlier [27], polymer **I-2** showed a maximum emission peak at 500 ± 20 nm in methanol that is similar to that of polymer **II-2** in the identical solvent. In cases of polymer **I-3** and **II-3**, they showed almost identical *λ*_em_ at 448 [40] and 446 nm in acetonitrile, respectively. Furthermore, **I-4** displayed *λ*_em_ peak at 440 and 442 nm when monitored at an excitation wavelength at 310 and 305 nm, in methanol and acetonitrile, respectively and, therefore, its *λ*_em_ peaks are close to polymer **II-4** in these two solvents. Similarly, the *λ*_em_ peaks of polymer **I-5** and **II-5** in methanol and acetonitrile were also essentially identical [35]. By changing the counterion from tosylate to macroion, PL spectra were not significantly different in identical solvents confirming as expected that chemical structures of the polymer backbones have more effect on the optical property than structures of counterions.

**Figure 9:**
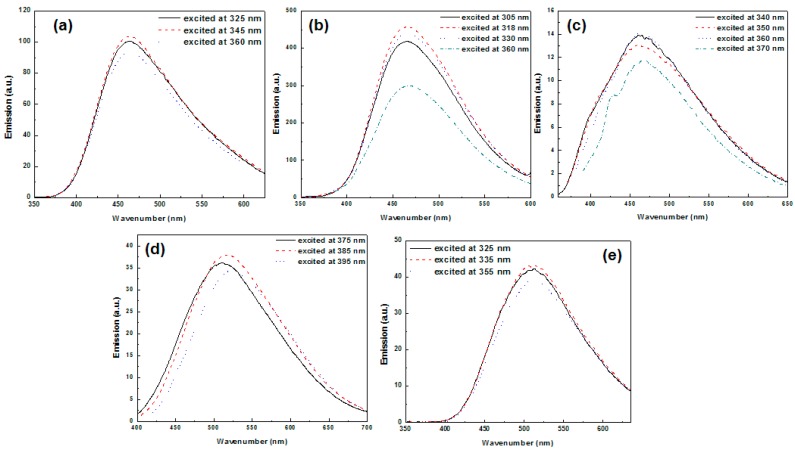
Emission spectra of polymers (**a**) **II-1**, (**b**) **II-2**, (**c**) **II-3**, (**d**) **II-4** and (**e**) **II-5** in thin films cast from methanol at various excitation wavelengths.

The films of polymers **II-1**–**II-5** were prepared from their respective solutions in methanol casting on quartz plates. The solid-state emission spectra of polymers are shown in Figure 9 and their *λ*_em_ peaks are summarized in Table 3. In thin film cast from methanol, polymer **II-1** showed a λ_em_ at 463 nm at excitation wavelengths 325, 345 and 360 nm, which was hypsochromically shifted by 34 nm when compared to its solution emission spectrum from methanol. Similarly, the methanol-cast films of polymers **II-2** and **II-5** exhibited hypsochromic shifts of 66 and 30 nm, respectively, when compared to their solution spectra. These features suggested that the bulky size of macrocounterion can interrupt the packing of these polymer chains in the solid state films cast from polar organic solvent resulting in less ordered structures. In contrast, the emission spectra of thin films of polymers **II-3** and **II-4** displayed bathochromic shifts of 10 and 30 nm, respectively, when compared to that of solution spectra. These polymer chains had more ordered structures in the solid states when cast from polar organic solvents compared to their solution spectra leading to bathochromic shifts. Note here that both intramolecular and intermolecular π−π interactions of chromophores of polymers are mainly responsible for the ordered structures, which usually cause both to shift *λ*_em_ bathochromically to a large extent as high as 100 nm or higher and to lower the quantum yields of light-emitting polymers in the solid state [51,52].

## 4. Conclusions

In summary, another series of poly(pyridinium) salts were prepared with a voluminous hydrophilic macrocounterion by metathesis reaction from the respective tosylate polymers in a common organic solvent. Their chemical structures were fully characterized by spectroscopic techniques. These polymers with poly(ethylene glycol) 4-nonylphenyl 3-sulfopropyl ether as new counterion led to water-soluble, room-temperature liquid or liquid-crystalline, and highly thermally stable polymers, representing thus an important and interesting new family of polymeric ionic liquids of importance for industrial and medical applications. The thermotropic LC properties of the ionic polymers were examined by experimental techniques including DSC, POM and SAXS, and only polymers **II-1** and **II-2** were found to be mesomorphic, displaying well-organized, highly segregated lamellar phases, whilst the other PILs **II-3**–**II-5** were obtained as room-temperature oily liquid, consequently to the nature of the various diamine connectors (rigid, semi-rigid, flexible) in the repeating units of the PILs. Due to their good solubility in water and common organic solvents, the optical properties of the polymers were studied by UV−Vis and photoluminescence spectrometer. In most of the solvents examined, they all emit blue light with the exception of one that emits green light. Three of the polymers emitted blue light and two of them emitted green light in the solid state. Both hypsochromic and bathochromic shifts in the solid state were observed depending on the microstructures of the polymeric backbones. This new method of preparing water-soluble, low-melting and thermally stable PILs can furnish a novel synthetic route to functionalization of poly(pyridinium salt)s that will broaden the applications of these materials.

## Figures and Tables

**Table 1:** Thermal properties of polymers **II-1**–**II-5** (determined during the second heating cycle).

Polymer	II-1	II-2	II-3	II-4	II-5
*T*_m/g_ (°C)	40 ^a^	29 ^c^	32 ^c^	34 ^c^	31 ^d^
*T*_i_ (°C)	200 ^b^	160 ^b^	−	−	−
*T*_d_^e^ (°C)	322	313	318	261	329

^a^ Glass to fluid LC phase transition temperature, as determined by Fisher-Jones melting point apparatus. ^b^ LC to the isotropic liquid phase transition temperature, *T*_i_, determined by SAXS. ^c^ *T*_g_, Glass to fluid LC phase or to the isotropic liquid phase transition temperature, as determined by DSC. ^d^ Melting temperature. ^e^ Onset temperature of the thermal decomposition, given at 5 wt.% loss, recorded under nitrogen.

**Table 2:** XRD data for polymers **II-1** and **II-2.**

Polymer	Temperature	*d*_meas._ (Å) ^a^	I ^b^	Indexation ^c^	*d*_theor._ (Å) ^a^	*A*_ru_ (Å^2^) ^d^
						h_ru_ (Å)
**II-1**	80 °C	63.9	VS, sh	1	64.07	73.5
		31.96	S, sh	2	32.04	3.44
		21.16	M, sh	3	21.36	
		15.85	M, sh	4	16.02	
		13.1	S, sh	5	12.81	
		10.7	W, sh	6	10.68	
		9.15	W, sh	7	9.15	
		8.02	W, sh	8	8.01	
		4.5	VS, br	−	*h*_ch_	
		4.35	M, sh	−	*h*_c_	
	140 °C	64	VS sh	1	64.15	76.2
		32	W, sh	2	32.08	3.57
		21.15	S, sh	3	21.38	
		13.03	VS, br	5	12.83	
		4.5	VS, br	−	*h*_ch_	
		4.36	M, sh	−	*h*_c_	
**II-2**	100 °C	67	VS, sh	1	66.95	71.5
		33.62	M, sh	2	33.47	3.57
		22.2	M, sh	3	22.31	
		16.74	W, sh	4	16.73	
		4.5	VS, br	−	*h*_ch_	

^a^*d*_meas._ and *d*_theor._ are the measured and calculated diffraction spacings, according to *d* = *d*_theor._ = (Σ00*l*Å × *d*_00*l*_)]/N*l*; *h*_ch_ maxima of the diffuse scattering due to lateral distances between molten tails; *h*_c_ lateral registry. ^b^ I corresponds to the intensity of the reflections (VS: very strong, S: strong, M: medium, W: weak; br and sh stand for broad and sharp). ^c^ *00l* Miller indices. ^d^ *L*_ru_ is the length of the monomeric repeat unit (*L*_ru_ = 21.36 and 20.03 Å, for **II-1** and **II-2**, respectively, obtained from reported crystalline structures). *V*_ru_ and *A*_ru_ = *V*_ru_/*d* are the volume and molecular area of the repeat unit, in the plane of the smectic layers. *h*_ru_ = *A*_ru_/*L*_ru_ is the average separation between the polymer backbones in the layer planes. The *h*_ru_ values indicate that the polymers’ backbones are orthogonal to the layers.

**Table 3:** Optical properties of poly(pyridinium) salts with macrocounterions, **II-1**–**II-5.**

Polymer	II-1	II-2	II-3	II-4	II-5
UV abs (nm) *^a^*	345	337	345	330	337
Band gap (eV) *^a^*	3.04	3.00	3.04	3.21	3.00
PL *λ*_em_ H_2_O (nm)	492	496	447	445	533
PL *λ*_em_ CH_3_OH (nm)	497	522	451	443	542
PL *λ*_em_ CH_3_CN (nm)	500	527	446	442	542
PL *λ*_em_ Acetone (nm)	487	−	446	442	534
PL *λ*_em_ THF (nm)	492	482	413	439	527
PL *λ*_em_ CHCl_3_ (nm)	463	−	421, 443	440	500, 520
PL *λ*_em_ film (nm) *^b^*	463	466	461	518	512

*^a^* Obtained from H_2_O. *^b^* Thin film cast from methanol.

## Supplementary Materials

The following are available online at https://www.mdpi.com/2073-4360/11/5/851/s1, Figure S1: FTIR spectra of polymers **II-1**–**II-5**, Figures S2–S5: ^1^H NMR spectra of polymers **II-1**, **II-3**–**II-5**, Figure S6: XRD patterns of polymers **II-3**–**II-5**, Figure S7: POM textures of polymer **II-1**, Figures S8–S12: Emission spectra of **II-1**–**II-5** in various organic solvents.
