# Supplementary material for: Thermotropic Liquid-Crystalline and Light-Emitting Properties of Poly(pyridinium) Salts Containing Various Diamine Connectors and Hydrophilic Macrocounterions
Source: Polymers (Basel). 2019 May 10;11(5):851. doi: 10.3390/polym11050851 (PMC6572631; doi:10.3390/polym11050851)
Supplement: Supplementary file 1 [file polymers-11-00851-s001.pdf]

Thermotropic Liquid-Crystalline and Light-Emitting Properties of Poly(pyridinium) Salts Containing Various Diamine Connectors and Hydrophilic Macrocounterions

Tae Soo Jo,[1] Haesook Han,[1] Pradip K. Bhowmik,*[1] Benoît Heinrich,[2] Bertrand Donnio,[2]

[1]*Department of Chemistry, University of Nevada at Las Vegas, 4505 Maryland Parkway, Box 454003, Las Vegas, NV 89154, USA*

[2]*Institut de Physique et Chimie des Matériaux de Strasbourg (IPCMS), UMR 7504, CNRS-Université de Strasbourg, BP 43, 23 rue de Loess, F-67034 Strasbourg Cedex 2, France*

*Corresponding author: Phone: (702) 895-0885. Fax: (702) 895-4072. E-mail: pradip.bhowmik@unlv.edu.

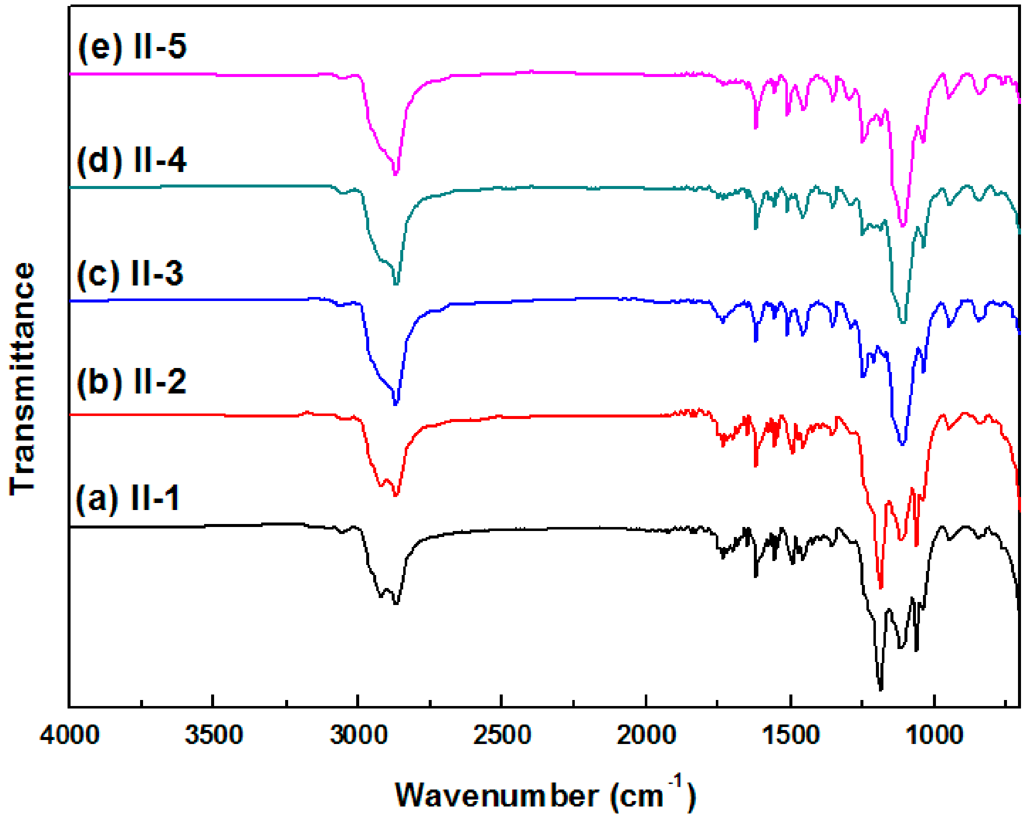

Figure S1. FTIR spectra of polymers **II-1**–**II-5** taken at room temperature.

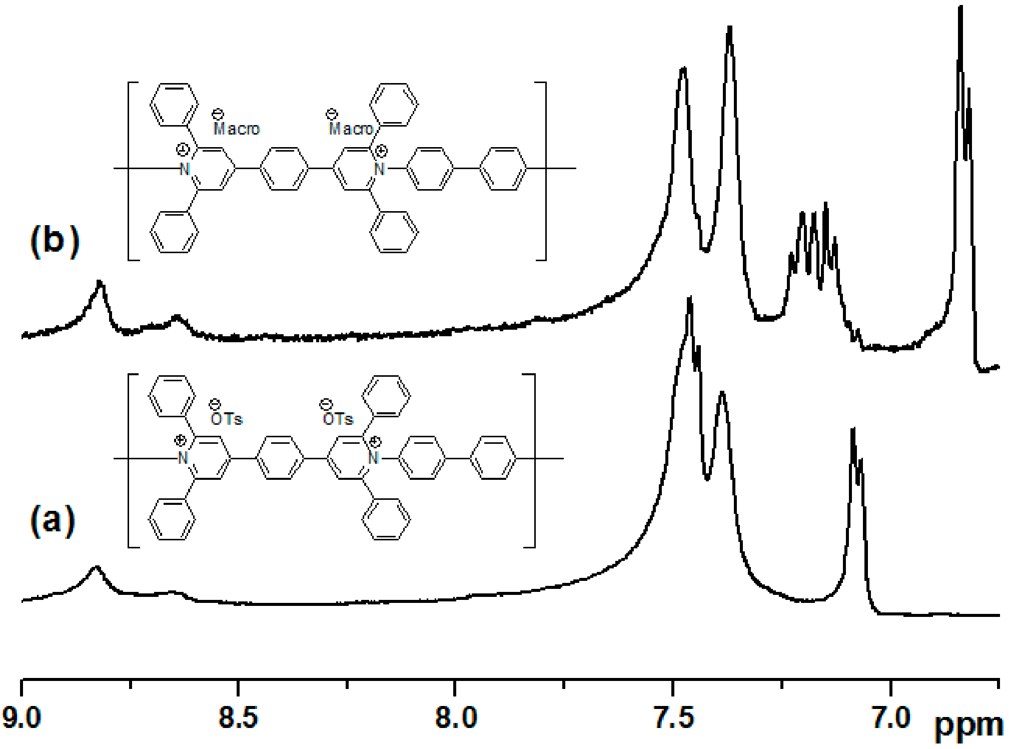

Figure S2. Expanded ^{1}H NMR spectra [delay time = 1 s, number of scans = 16] of polymers (a) **I-1** and (b) **II-1** [10 mg/mL in d_6-DMSO at 25 ^{o}C].

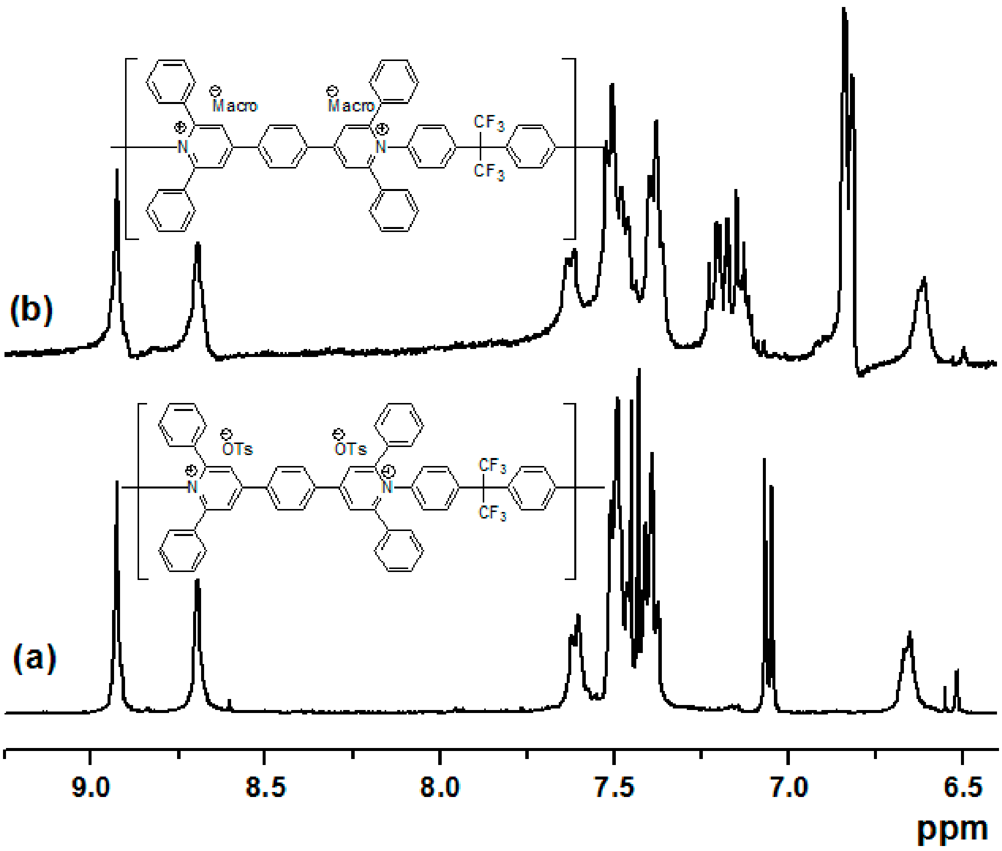

Figure S3. Expanded ^{1}H NMR spectra [delay time = 1 s, number of scans = 16] of polymers (a) **I-3** and (b) **II-3** [10 mg/mL in d_6-DMSO at 25 °C].

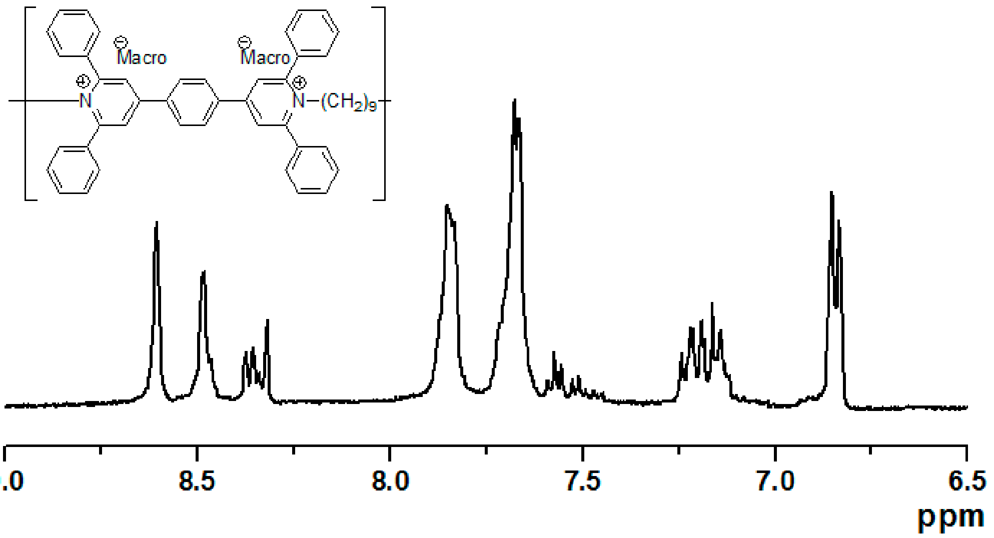

Figure S4. Expanded ^1H NMR spectrum [delay time = 1 s, number of scans = 16] of polymer **II-4** [10 mg/mL in d_6-DMSO at 25 ^oC].

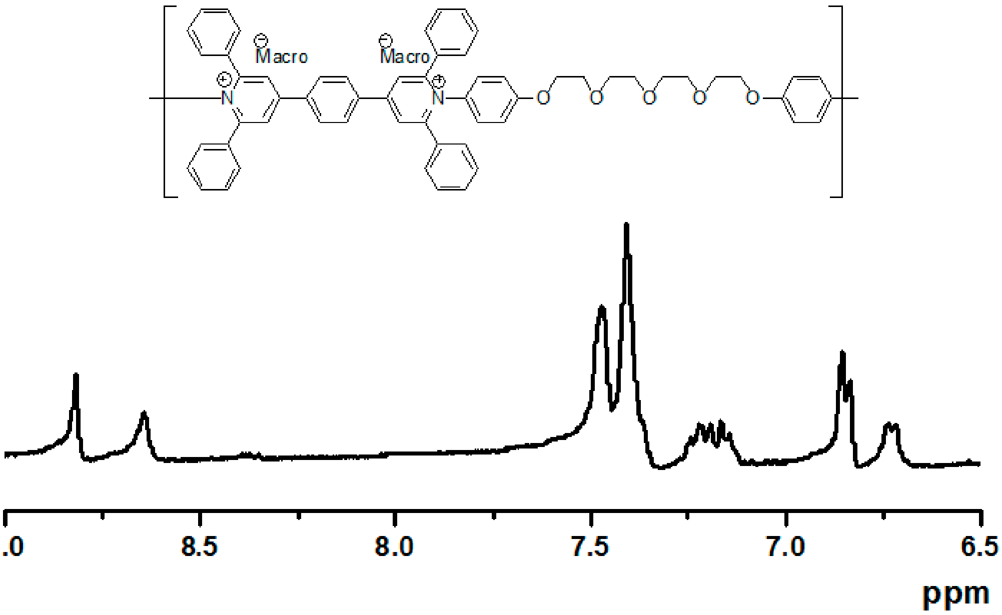

Figure S5. Expanded ^1H NMR spectrum [delay time = 1 s, number of scans = 16] of polymer **II-5** [10 mg/mL in d_6-DMSO at 25 ℃].

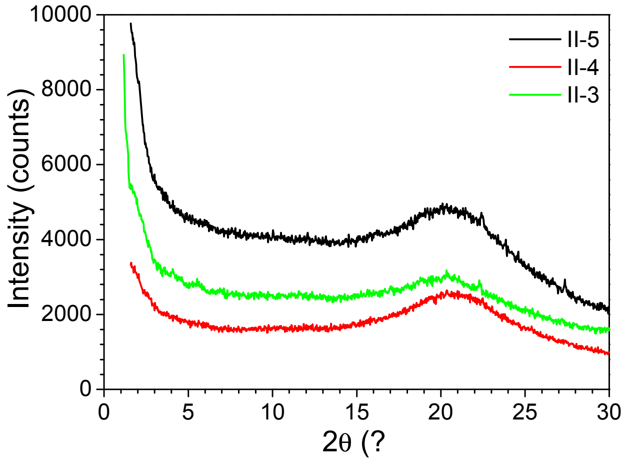

Figure S6. Small-angle X-ray scattering patterns of the amorphous phases of polymers **II-3–II-5** registered at room temperature. The strong halo in the wide-angle range of the diffractogram, with a maximum at 4.4–4.6 Å, was readily assigned to the overlapping distances between molten aliphatic chains and semi-rigid backbones.

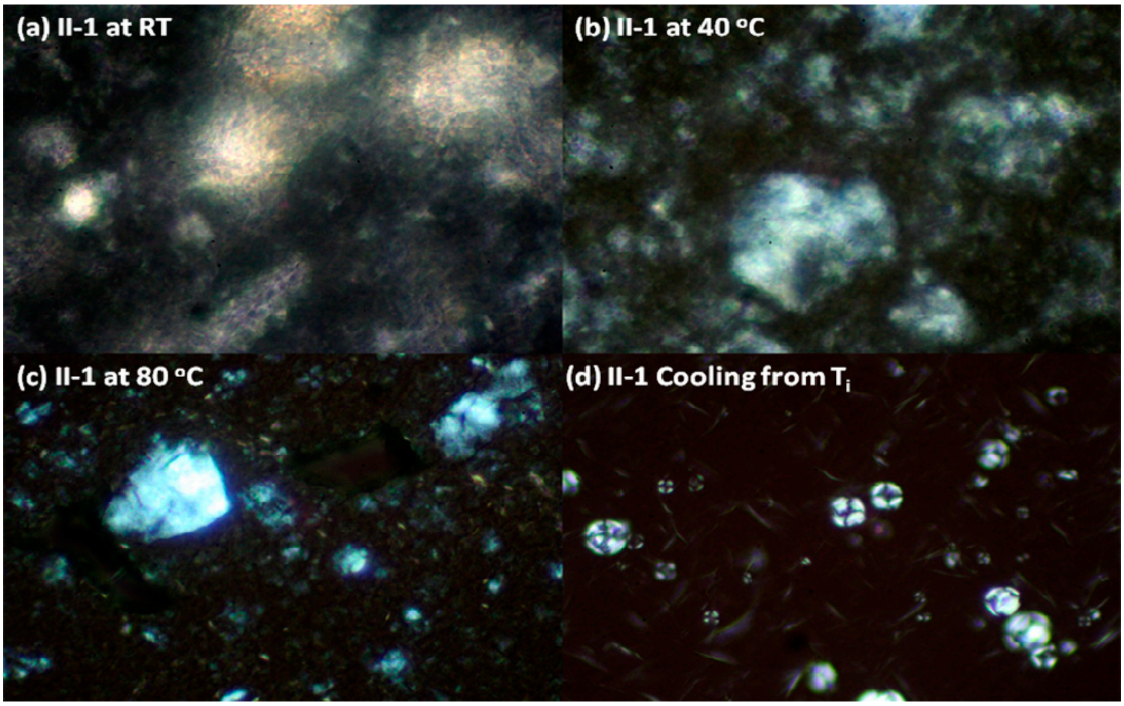

Figure S7. Photomicrographs of polymers **II-1** at room temperature (crystalline phase) and lamellar phase under crossed polarizers exhibiting thermotropic LC phase (magnification 400x).

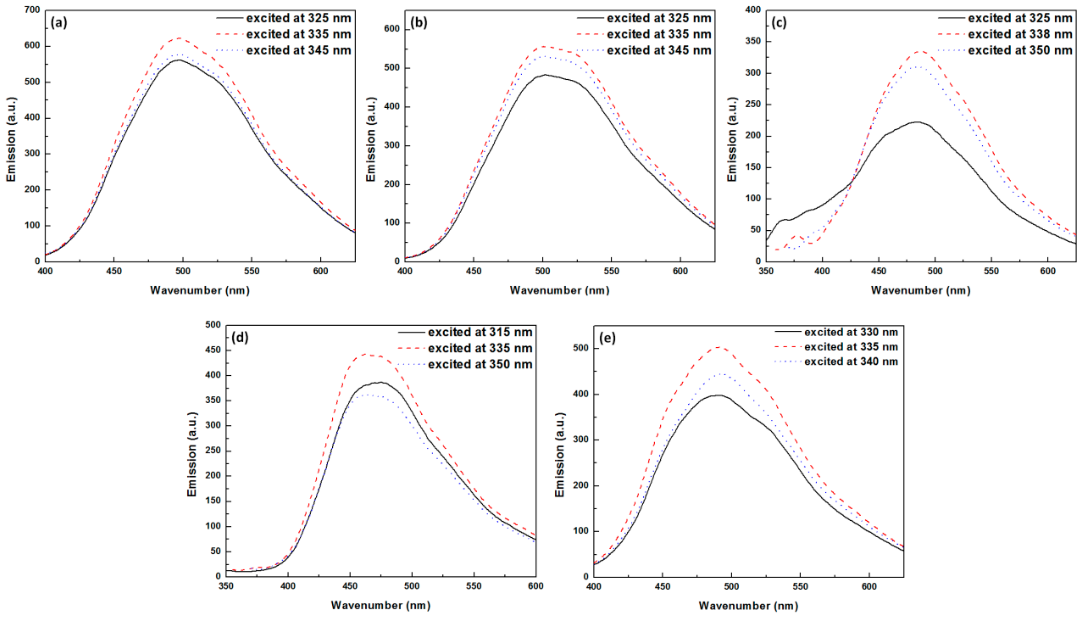

Figure S8. Emission spectra of polymer **II-1** in (a) MeOH, (b) CH_3CN, (c) acetone, (d) $CHCl_3$ and (e) THF at various excitation wavelengths.

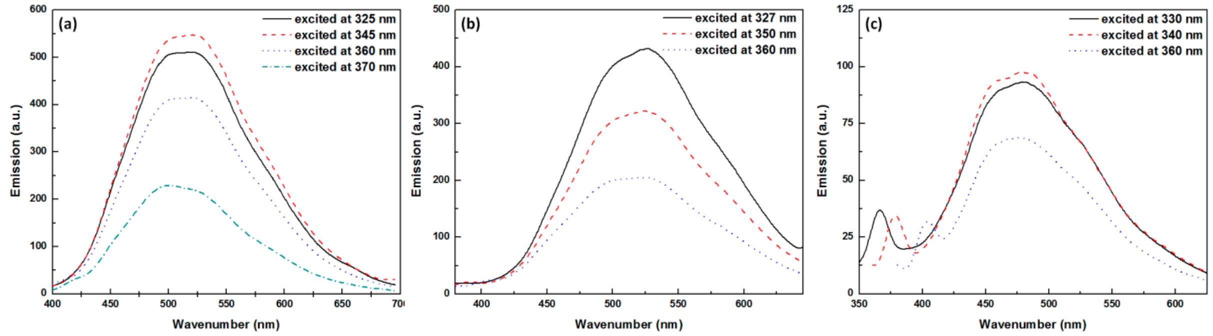

Figure S9. Emission spectra of polymer **II-2** in (a) MeOH, (b) CH_3CN, and (c) THF at various excitation wavelengths.

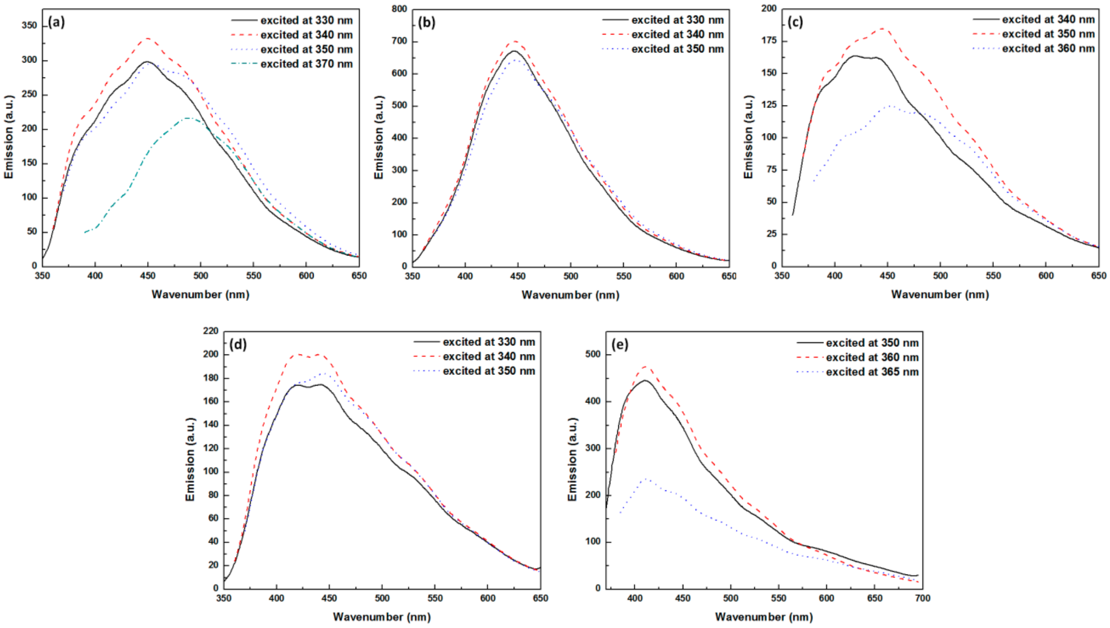

Figure S10. Emission spectra of polymer **II-3** in (a) MeOH, (b) CH_3CN, (c) acetone, (d) $CHCl_3$ and (e) THF at various excitation wavelengths.

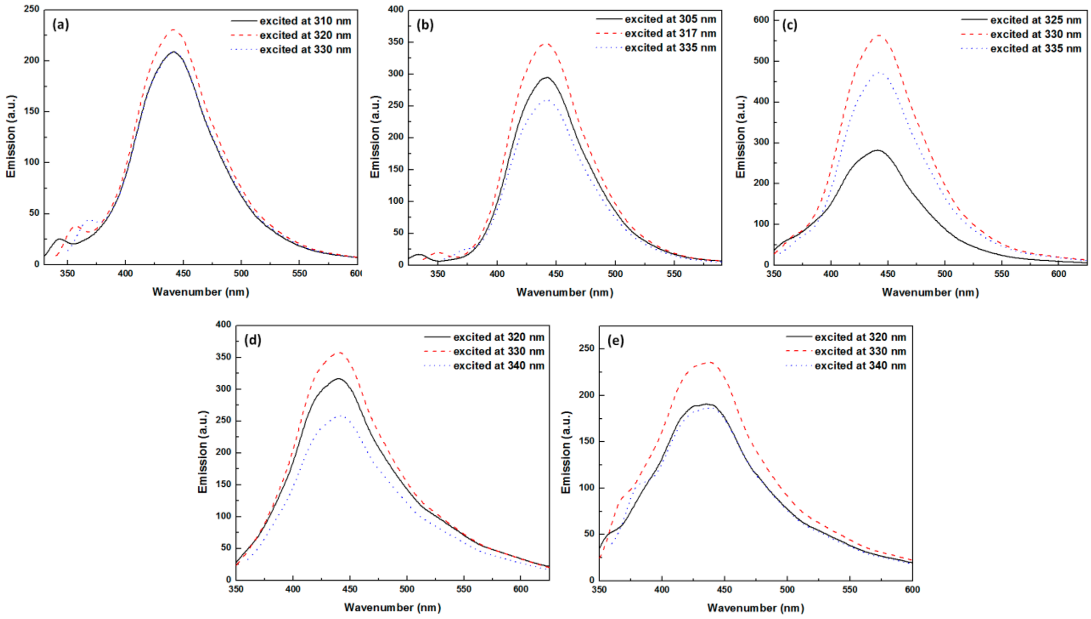

Figure S11. Emission spectra of polymer **II-4** in (a) MeOH, (b) CH_3CN, (c) acetone, (d) $CHCl_3$ and (e) THF at various excitation wavelengths.

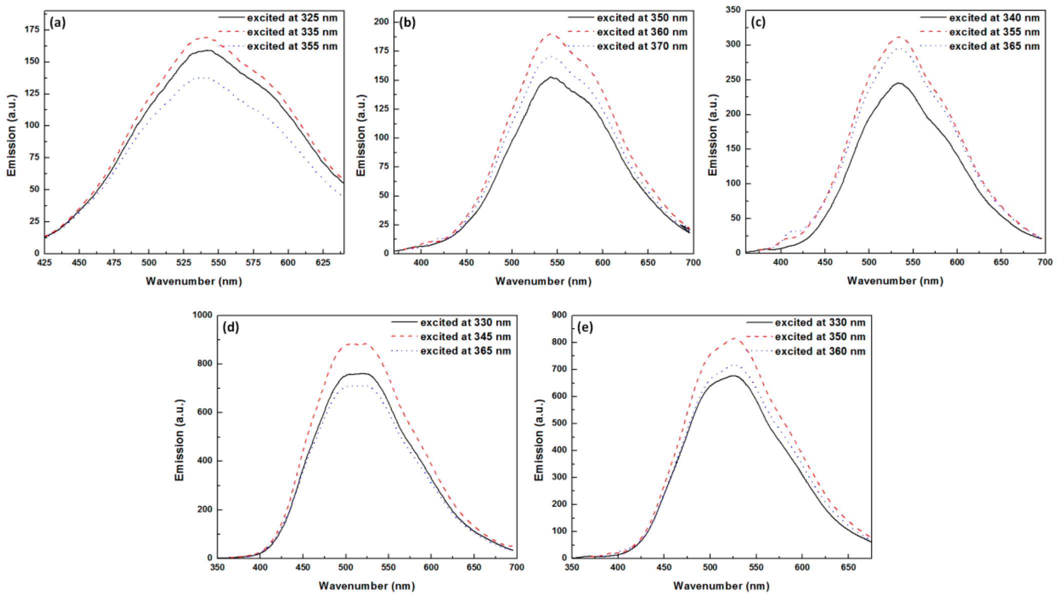

Figure S12. Emission spectra of polymer **II-5** in (a) MeOH, (b) CH_3CN, (c) acetone, (d) $CHCl_3$ and (e) THF at various excitation wavelengths.
